# Upright Balance Control in Individuals with Cervical Myelopathy Following Cervical Decompression Surgery: A Prospective Cohort Study

**DOI:** 10.1038/s41598-020-66057-y

**Published:** 2020-06-25

**Authors:** Chih-Hsiu Cheng, Dar-Ming Lai, Phooi Yee Lau, Shwu-Fen Wang, Andy Chien, Jaw-Lin Wang, Wei-Li Hsu

**Affiliations:** 1 School of Physical Therapy and Graduate Institute of Rehabilitation Science, College of Medicine, Chang Gung University, Taoyuan City, Taiwan; 2 Department of Surgery, National Taiwan University Hospital, Taipei, Taiwan; 3 School & Graduate Institute of Physical Therapy, College of Medicine, National Taiwan University, Taipei, Taiwan; 4 Physical Therapy Center, National Taiwan University Hospital, Taipei, Taiwan; 5 Department of Physical Therapy & Graduate Institute of Rehabilitation Science, China Medical University, Taichung, Taiwan; 6 Institute of Biomedical Engineering, National Taiwan University, Taipei, Taiwan

**Keywords:** Rehabilitation, Neurosurgery

## Abstract

Patients with cervical myelopathy may manifest impairments in functional activities and balance control caused by compression of the spinal cord. The objective of the current study was to determine long-term changes in the upright balance control of patients with cervical myelopathy who had undergone cervical decompression surgery. This is a prospective cohort study from the preoperative phase to 3 months, 6 months, and 1 year postsurgery. Fifty-three patients with cervical myelopathy were recruited for the cervical myelopathy group and 22 age-matched healthy controls were recruited for the control group. Functional assessments including Japanese Orthopedic Association Cervical Myelopathy Evaluation Questionnaire-Lower Extremity Function (JOACMEQ-LEF) and 10-second step test; as well as balance assessments including postural sway (center-of-pressure: COP) were performed for both groups. The JOACMEQ-LEF (p = 0.036) scores of the myelopathy group improved postoperatively, and a significant decrease in COP variables of postural sway was observed. The upright posture was less stable in the myelopathy group than in the control group (p < 0.05) both before and after surgery. The effect size and standard response mean of the COP variables ranged from −0.49 to 0.03 at 3 months, 6 months, and 1 year postsurgery. The upright balance control had improved significantly 6 months after decompression surgery. However, the balance control of the patients who had undergone decompression surgery remained less stable than that of the age-matched healthy controls. Balance training should be initiated before 6 months postsurgery to accelerate balance control recovery in patients with cervical myelopathy.

## Introduction

Cervical myelopathy is a condition caused by compression of the spinal cord and can cause direct injury to nerves^1^. Symptoms of cervical myelopathy, including loss of hand dexterity, standing imbalance, gait impairment, sensory loss, and bladder dysfunction, correspond to the level of cervical spine injury^2^.

Cervical decompression surgery is a common invasive intervention for cervical myelopathy^3^. The effectiveness of decompression surgery is usually evaluated based on functional outcomes, which are related to the effects of clinical manifestations on daily activities^4^. Although decompression surgery can alleviate the symptoms of myelopathy, surgical outcomes may be affected by age and symptom duration^5^. Long symptom duration may be associated with poor outcomes in functional activities that require stability in upright positions such as standing and walking^6^.

Gait parameters such as velocity, step length, cadence, stance phase, and single-stance phase have been reported to improve after decompression surgery^7,8^. However, in a recent study, no measured spatiotemporal parameters changed significantly postsurgery, except for electromyography findings^9^. Inconsistent results have been reported regarding postoperative effects on gait in patients with myelopathy. Moreover, increased signal intensity in T2-weighted cervical spine magnetic resonance images was correlated with impaired gait performance but not with scores of the modified Japanese Orthopedic Association (JOA) scale and Nurick scale^10^. Therefore, functional parameters other than gait such as upright balance control, which might be correlated with disease severity^11^, should be considered for postsurgical assessment.

Center-of-pressure (COP) movement is a simple biomechanical variable that can reflect an individual’s ability to maintain upright balance control^12,13^. COP movement parameters such as sway area, mean velocity, and range of traveling can quantify postural sway during movement^14,15^. Moreover, collecting data for COP movement is simple, and thus such data can easily be used in clinical settings. The present study investigated long-term changes in upright balance control (i.e., COP movement) in patients with myelopathy who had undergone cervical decompression surgery.

## Methods

### Study design

This observational prospective cohort study study determined improvements in upright balance control in patients with cervical myelopathy at 3 months, 6 months, and 1 year postsurgery. This study is part of as part of an ongoing research register in ClinicalTrial.gov (Identifier: NCT03396055). The study was approved by the Research Ethics Committee of National Taiwan University Hospital (201505093RIN).

### Study procedure

**Figure 1:**
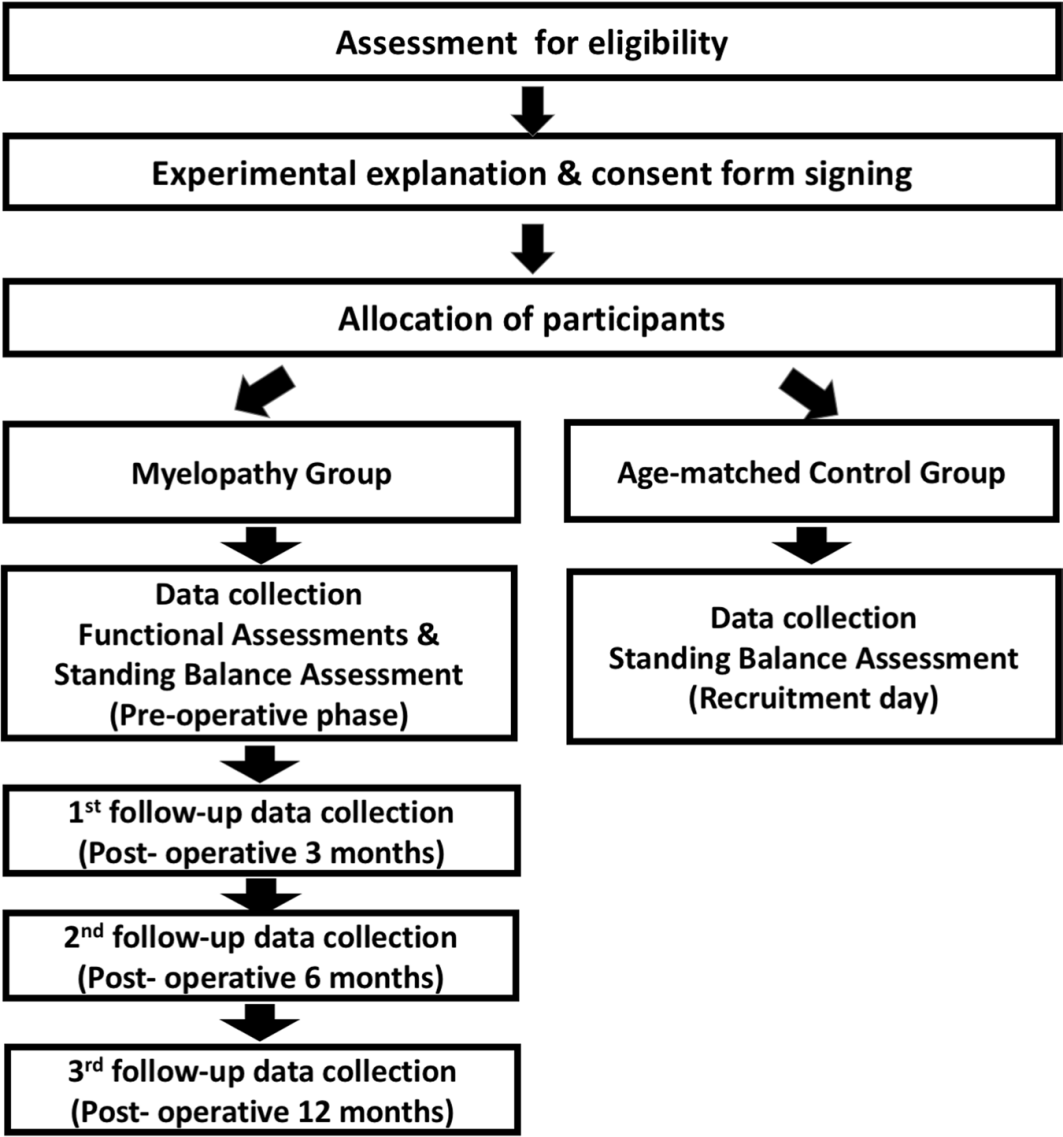
Flowchart of the study procedure.

The study flowchart is shown in Fig. 1. Patients with myelopathy and age-matched healthy controls were assessed for eligibility. The study purpose was explained to all recruited participants, and written informed consent was obtained from all participants.

### Participant recruitment

Fifty-three patients with myelopathy were recruited. The inclusion criteria for the myelopathy group were age between 20–80 years, presence with one or more neurological sign/symptom of cervical myelopathy and a diagnosis of cervical myelopathy according to relevant imaging examination. All patients in the myelopathy group were assessed by the same neurosurgeon to determine the requirement of decompression surgery. Participants were excluded if the participants were not able to stand upright for 1 minute, were unable to communicate or follow instructions, were unsuitable candidates for cervical decompression surgery because of other medical conditions, had traumatic spinal injury, had previous neurological dysfunction of the central nervous system (CNS), had recent musculoskeletal injury to the lower extremities, had vestibular dysfunction, or had infection or metastasis in the spine or lower extremities.

Twenty-two age-matched healthy controls were recruited. The inclusion criteria for the healthy control group were age between 20–80 years and absence of neck or back pain, severe musculoskeletal injury to the lower extremities or spine, vestibular dysfunction, and neurological dysfunction.

### Functional assessments

Functional assessments were conducted in the myelopathy group. The JOA Cervical Myelopathy Evaluation Questionnaire (JOACMEQ) is a self-reported instrument used to evaluate the severity of cervical myelopathy. The total score of each item on this scale is generally high because patients’ self-reported conditions usually improve following treatment^16^. In this study, the JOACMEQ-Lower Extremity Function (JOACMEQ-LEF), which examines an individual’s ability to walk on a flat surface, stand on one leg, climb stairs, bend forward, kneel or stoop, and walk for more than 15 minutes, was used^16,17^.

The 10-second step test is another instrument to evaluate the severity of cervical myelopathy^18^. The participants were asked to take a step by lifting the thighs parallel to the floor in the same place without support at maximum speed^18^. The number of steps performed in 10 seconds was counted. Each participant was asked to perform the test at maximum speed^18^. For safety purposes, the examiner supervised all participants to prevent fall incidents.

### Upright balance assessment

The upright balance assessment was conducted at four time points for the myelopathy group and on the recruitment day for the control group. Participants were asked to stand on a force platform (Kistler 9286 A, Kistler Instrumente AG, Winterthur, Switzerland) for 30 seconds at a sampling rate of 1000 Hz in each standing trial. All participants adopted a neutral stance (feet shoulder width apart) with eyes open and eyes closed. The same standing balance assessment was conducted for the remaining 37 participants on a different force platform (AMTI OR6, Advanced Medical Technology Inc., Watertown, MA, USA) for 30 seconds at a sampling rate of 1000 Hz. The participants were allowed to rest if they felt tired or soreness in the legs.

### Data processing

Force platform signals were converted from analog to digital at a sampling rate of 1000 Hz. LabVIEW (National Instruments Corp., Austin, TX, USA) software was used to compute COP movement based on ground reaction force and moment in anteroposterior (AP) and mediolateral (ML) directions. Subsequently, collected data were processed and filtered through a second-order Butterworth low-pass filter of 5 Hz by using Matlab R2010a software. COP movement was further analyzed for time-domain measures, namely COP excursion, sway velocity, and sway area^14^. The COP sway area is defined as the area of the 95% of all points on the COP path. COP sway velocity is defined as the mean average velocity of the COP sway. The COP range is defined as the greatest distance between any two points on the COP movement path in anterior-posterior (AP) and medial-lateral (ML) direction. The root mean square (RMS) distance of COP was defined as RMS value of the resultant distance time series, which was also calculated in AP and ML direction respectively.

COP displacement reflects how the body moves to maintain balance stability. The previous study^14^ illustrated the time domain measures of COP movement among healthy young adults (21–35 years old) and healthy elderly (66–80 years old) in eyes open and eyes closed condition respectively. Besides, COP movement was used to evaluate balance performance in patients with cervical^19–21^ and lumbar^13^ disorder. However, the comprehensive time-domain COP normative data has not been published before for patients myelopathy for different level of severity.

### Statistical analysis

The sample size in this study was determined by G* Power Software version 3.1 based on the pilot study on the COP data in patients with myelopathy group. Statistical analysis was performed using PASW Statistics 18 for Macintosh (SPSS, Chicago, IL, USA). Nonparametric tests were used for data analysis because functional outcomes and COP movement variables were not normally distributed. A p value of less than 0.05 (alpha, α) was considered statistically significant.

Differences in functional outcomes (JOACMEQ-LEF and 10-second step test) and in COP variables in the four phases served as the main effect and were examined using the Friedman test. If the main effect of phase difference was detected, pairwise comparisons of variables between the phases were performed using the Wilcoxon signed-rank test with Bonferroni adjustment. The difference in COP variables between the myelopathy and age-matched control groups over the four phases was determined using the Mann–Whitney U test.

## Results

**Figure 2:**
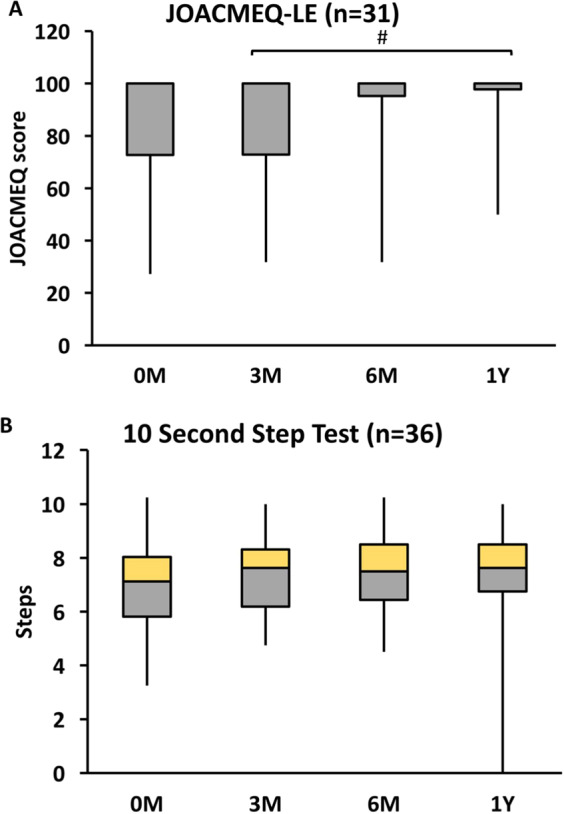
Comparison of Japanese Orthopaedic Association Cervical Myelopathy Evaluation Questionnaire-lower extremities function (JOACMEQ-LEF) and 10 second step test after 3 months, 6 months and 1 year of surgery (preoperative phase as baseline). 0 M: preoperative phase; 3 M: postoperative 3 months; 6 M: postoperative 6 months; 1Y: postoperative 1 year. *Significant difference between 0 M and 1Y (p < 0.05/6 after Bonferroni’s adjustment). ^#^Significant difference between 3 M and 1Y (p < 0.05/6 after Bonferroni’s adjustment).

The basic of information of the participants are illustrated in Table 1. Figure 2 shows the results of the Friedman test for the phase factor in functional assessments (i.e., the JOACMEQ-LEF and 10-second step test). We observed a significant difference among the phases in JOACMEQ-LEF scores (p = 0.036). The results of pairwise comparison revealed a significant difference in the JOACMEQ-LEF between 3 months and 1 year postsurgery (p = 0.002).

**Table 1:** Descriptive characteristic of myelopathy group with surgery and healthy age-matched control group.

	Myelopathy Group	Age-matched Control Group	p value
Subject Number (Male: Female)	53 (40:13)	22 (6:16)	—
Age (years)	55.50 ± 9.63	56.41 ± 9.66	0.861
Height (m)	1.65 ± 0.08	1.62 ± 0.07	0.087
Weight (kg)	68.73 ± 11.28	63.32 ± 9.90	0.054
BMI (kg/m^2^)	25.19 ± 3.33	24.24 ± 3.32	0.268
**Surgical Method**			
ACDF	24	NA	
Laminoplasty	15	NA	
Arthroplasty	1	NA	
ACDF + Arthroplasty	12	NA	
Other	1	NA	

BMI: body mass index; ACDF: anterior cervical discectomy and fusion; NA: not available.

**Figure 3:**
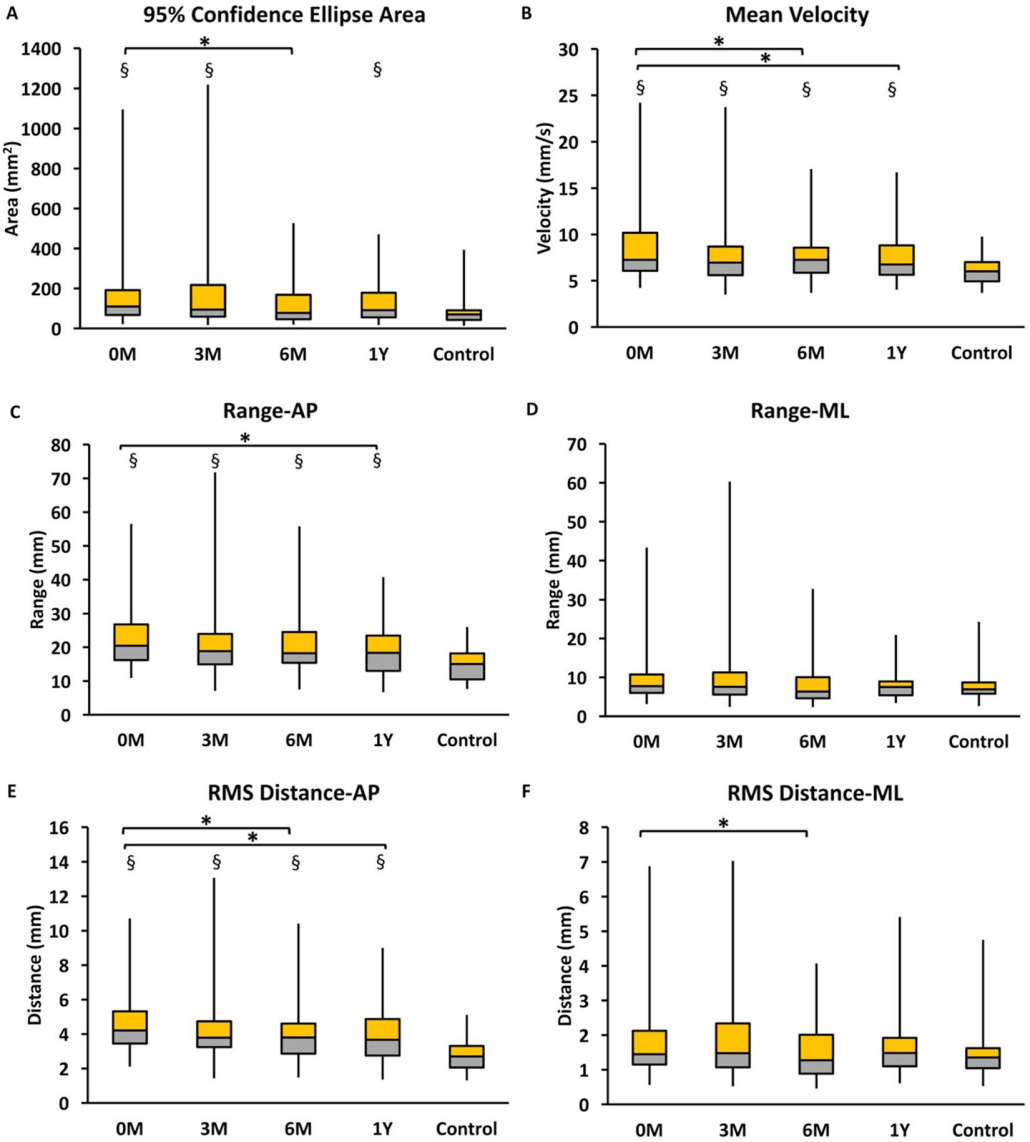
Comparison between phases and groups in COP variables during eyes-open stance in (**A**) 95% confidence ellipse area, (**B**) mean velocity, (**C**) range-AP, (**D**) range-ML, (**E**) RMS distance-AP and (**F)** RMS distance-ML. 0 M: myelopathy group at preoperative phase; 3 M: myelopathy group at postoperative 3 months; 6 M: myelopathy group at postoperative 6 months; 1Y: myelopathy group at postoperative 1 year; Control: age-matched control group. *Significant difference between phases of surgery (p < 0.05). ^§^Significant difference between myelopathy group and age-matched control group (p < 0.05).

**Figure 4:**
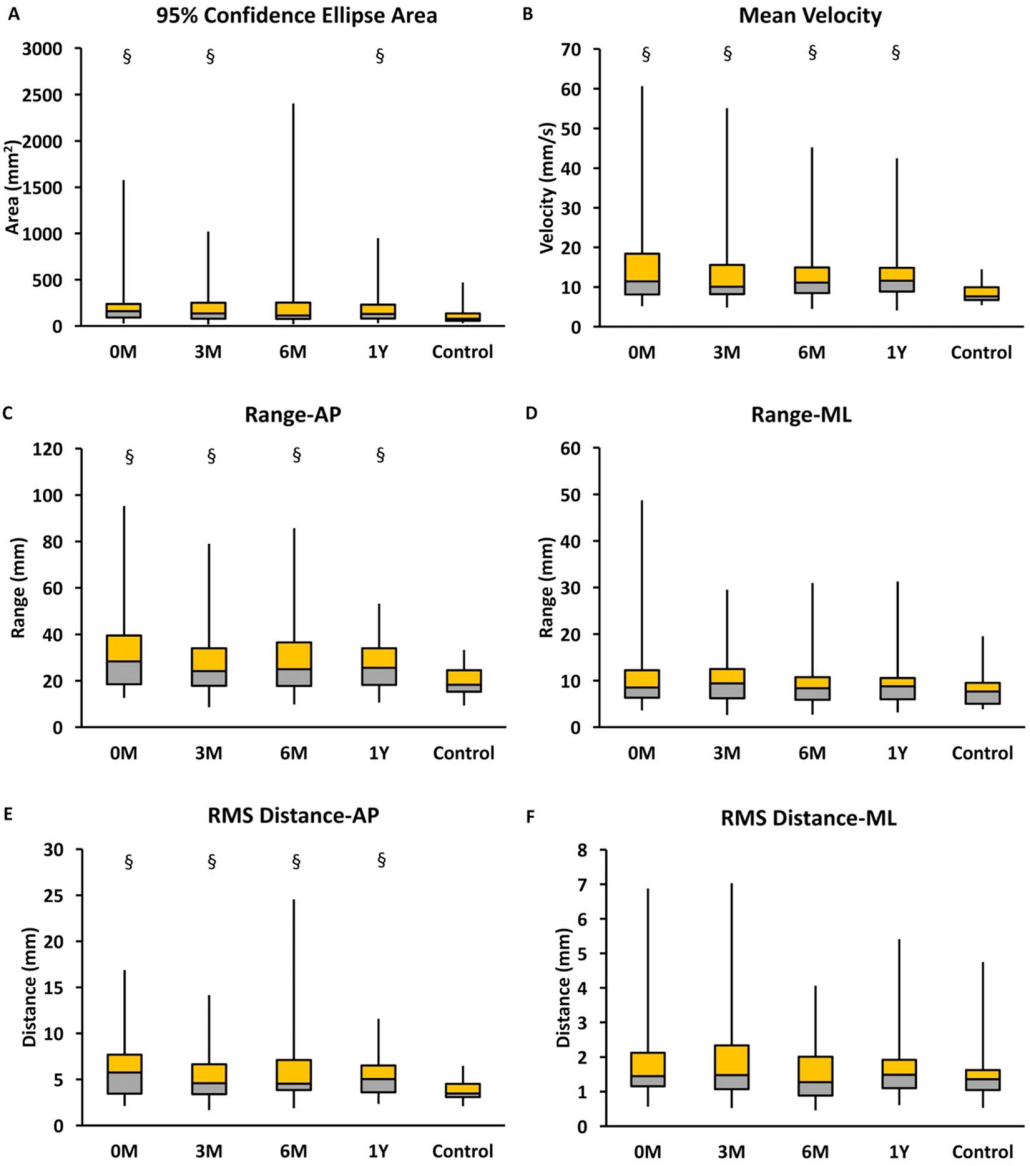
Comparison between phases and groups in COP variables during eyes-closed stance in (**A**) 95% confidence ellipse area, (**B**) mean velocity, (**C**) range-AP, (**D**) range-ML, (**E**) RMS distance-AP and (**F**) RMS distance-ML. 0 M: myelopathy group at preoperative phase; 3 M: myelopathy group at postoperative 3 months; 6 M: myelopathy group at postoperative 6 months; 1Y: myelopathy group at postoperative 1 year; Control: age-matched control group. *Significant difference between phases of surgery (p < 0.05). ^§^Significant difference between myelopathy group and age-matched control group (p < 0.05).

Figure 3 presents the results of the Friedman test for the phase factor of COP variables in the eyes-open stance. Significant differences were found in all COP variables among the phases of decompression surgery in the eyes-open stance (95% confidence ellipse area: p = 0.022; mean velocity: p = 0.019; range-AP: p = 0.007; range-ML: p = 0.017; RMS distance-AP: p = 0.023; RMS distance-ML: p = 0.028). The pairwise comparison results revealed significant differences in the 95% confidence ellipse area (p = 0.006), mean velocity (p = 0.008), and RMS distance-AP (p = 0.004) between the preoperative phase and 6 months postsurgery. Moreover, the pairwise comparison results also showed significant differences in the mean velocity (p = 0.004), range-AP (p = 0.001), and RMS distance-AP (p = 0.004) between the preoperative phase and 1 year postsurgery. However, the COP variables did not differ significantly among the phases of decompression surgery in the eyes-closed stance (Fig. 4).

Figures 3 and 4 show the results of the Mann–Whitney U test for the four phases of surgery in the eyes-open and eyes-closed stances, respectively, for the control and myelopathy groups. The 95% confidence ellipse area differed significantly between the control and myelopathy groups in the preoperative phase and at 3 months and 1 year postsurgery (p < 0.05) in the eyes-open and eyes-closed stances. The mean velocity, range-AP, and RMS distance-AP differed significantly among the four phases of surgery (p < 0.05) in the eyes-open and eyes-closed stances between the control and myelopathy groups.

## Discussion

The present study investigated changes in upright balance control in patients with myelopathy who had undergone decompression surgery. The findings of this study indicated that upright balance control had improved at 6 months postsurgery. However, compared with the age-matched healthy controls, the upright posture of the patients with myelopathy remained less stable even after surgery.

The findings of the JOACMEQ-LEF, a subjective assessment, showed improvements at 1 year postsurgery which was in line with those of previous studies^22–24^. However, the results of the 10-second step test, an objective functional assessment, did not show significant improvements after surgery. Movement in the 10-second step test depends on the loading and unloading of the lower extremities in the ML plane by the hip abductor or adductor^25^. In our study, the patients with myelopathy exhibited AP balance control impairment. Thus, the 10-second step test may not be able to reflect the recovery in these patients.

In our study, upright balance control exhibited gradual improvement in the patients with myelopathy at 6 months and 1 year postsurgery. The slow healing of injured neural tissues in the spinal cord may have delayed the improvement of upright balance control in the early stage of the postoperative phase. A previous animal study on spinal cord injury reported that sprouting of corticospinal tract fibers occurred between 3 weeks and 3 months after injury, with penetration of the axons of this tract into the lesion matrix occurring over a long period^26^. Patients are usually asked to wear a neck collar to limit movement in their necks for the first 3 months after surgery. Patients may also become postoperatively inactive because of fear of movement in the initial 3 months postsurgery^27^. Thus, improvement of balance steadiness started after the termination of the immobilization phase. Postoperative motor relearning and balance training should be initiated before 6 months postsurgery to accelerate balance control recovery. Patients with shorter periods between symptom onset and rehabilitation are expected to achieve greater improvements in functional outcomes^28^.

In our study, upright balance control gradually improved in the patients with myelopathy in the eyes-open stance only. Sensorimotor impairment should be considered because ascending (sensory) and descending (motor) fibers in the spinal cord may be injured or damaged after compression^20^. Patients with myelopathy may be more mobile and active in daily functions performed in an upright position with eyes open. To maintain balance stability, various sensory inputs may be reweighted by the CNS^29,30^. In this study, visual inputs may have outweighed the proprioceptive input of the lower extremities to regulate balance control in the patients with myelopathy during the eyes-open stance^31^. Delayed recovery of proprioception may increase the balance sway of patients with myelopathy during the eyes-closed stance because of inefficient sensory integration for balance control^32^. These factors explain the delayed postoperative recovery of balance stability in the patients in the eyes-closed stance.

Either before or after decompression surgery, the balance steadiness of the patients with myelopathy (particularly in the AP direction) was less stable than that of the age-matched healthy controls in both the eyes-open and eyes-closed stances. Several assumptions can explain the poor upright balance control observed in the patients with myelopathy.

First, the sensorimotor deficit may not be completely recovered at 1 year postsurgery. Decompression surgery expanded the transverse area of the cervical canal for reversible cord injury and facilitated the morphological recovery of injured nerve tissues^33^. However, various pathological progressions might decrease the viscoelasticity of the cervical spinal cord, thereby indicating the delay and low degree of recovery^33^. The delayed recovery of injured cord tissues may impair ankle muscles for dorsiflexion and plantarflexion control, which are responsible for AP balance control, during upright standing^12,34^.

Second, some patients with myelopathy may have irreversible injury to a part of the spinal cord, particularly the corticospinal tract, after undergoing prolonged compression^35,36^. Surgical outcomes of decompression surgery are reportedly affected by the severity of histological changes secondary to spinal cord compression^35,36^. Histological changes in neuronal cells such as gliosis^35^, microcavities^35^, demyelination^35^, myelomalacia^36,37^, spongiform changes^36,37^, and necrosis^36,37^ may be caused by severe spinal compression or repetitive microtrauma in the spinal cord^38^. Irreversible pathological alterations in the spinal cord lead to permanent impairment of the proprioception of the lower extremities and the control of distal muscles (e.g., the tibialis anterior). Thus, a patient’s recovery may not progress further after 1 year postsurgery.

Third, cortical reorganization plasticity may trigger the recovery of upright balance control^39,40^, because decompression surgery can terminate the injury mechanism in the spinal cord. However, patients who undergo decompression surgery may be observed lack of steadiness or develop a fear of falling because of their adaptation to the previous compensatory strategy as a “safer way” to stand^9,41^. Patients with lumbar spinal fusion may exhibit a shorter forward reach distance because of fear avoidance^42,43^; this may cause them to stand in a compensatory pattern, even when the sensorimotor spinal pathway is recovering. Consequently, although the sensorimotor spinal pathway is recovering, the recovery of cortical reorganization of patients may be slowed because of a lack of task-specific practice^39,44^.

Finally, the adaptive or compensatory mechanism may be present in the musculoskeletal system^45^. The cervical muscle dysfunction and the pathological changes in the spinal tract may trigger an altered movement strategy in the lower extremities to compensate for sensorimotor dysfunction^41,46^. For instance, to maintain balance stability, the delayed antagonist reaction of the tibialis anterior^20^ may trigger corrective responses in the trunk and proximal joint^47^. Fatigability with incomplete spinal cord lesions may be associated with changes in muscle properties and characteristics of poor control such as muscle weakness, muscle atrophy, and delayed activation^48^ in the distal end of the lower extremities. The habitual compensatory pattern may persist after surgery if patients have not learned the correct movement pattern.

The findings of the current study demonstrated the progression of functional outcomes and upright balance control in individuals with cervical myelopathy who had undergone cervical decompression surgery up until 1 year postsurgery. The findings provide biomechanical evidence of balance control after decompression surgery. Incomplete recovery of upright balance control indicates that early mobilization should be initiated as soon as possible after surgery^49^. Postoperative rehabilitation should be initiated after termination of the postoperative neck immobilization phase. Motor relearning programs and customized balance training should be introduced in postoperative rehabilitation as soon as possible. In addition, COP variables can be used to examine the upright balance control of patients with myelopathy in clinical settings because balance control indicates recovery of the sensorimotor function^3^.

### Study limitations

First, the lifestyles and exercise habits of our participants were not controlled for and may have resulted in various surgical outcomes. Second, the assessment was performed only once in the control group without continuous follow-up; thus, the aging effect in the control group within 1 year could not be excluded from this study.

## Conclusions

The upright balance control of the patients with cervical myelopathy had improved 6 months after cervical decompression surgery. This postoperative improvement of upright balance control was observed only during the eyes-open stance. The upright posture of the patients with myelopathy remained less stable than that of the age-matched healthy controls after surgery. This result may be attributed to incomplete recovery of cervical spinal cord injury, permanent damage to spinal cord cells, and ongoing cortical reorganization and musculoskeletal adaptation changes in the patients who had undergone decompression surgery. Balance training could be initiated as early as 3 months postsurgery to accelerate balance control recovery in patients with cervical myelopathy.
